# Emergence and Spread of Antimicrobial-Resistant Pathogens in an Era of Globalization

**DOI:** 10.1155/2012/286703

**Published:** 2012-10-16

**Authors:** Abiola C. Senok, Giuseppe A. Botta, Olusegun O. Soge

**Affiliations:** ^1^Department of Pathology and Pharmacology, College of Medicine, Alfaisal University, P.O. Box 50927, Riyadh 11533, Saudi Arabia; ^2^Section of Microbiology, Department of Medical and Morphological Research, Medical School, University of Udine, 33100 Udine, Italy; ^3^Department of Global Health, University of Washington, Seattle, WA, USA

In recent years, we have witnessed an increased emergence of antimicrobial-resistant (AMR) pathogens. In this era of globalization, international travel has been implicated as a significant risk factor for the acquisition of infections with multidrug-resistant bacteria, including *Acinetobacter* spp., methicillin-resistant *Staphylococcus aureus*, vancomycin-resistant enterococci, hypervirulent *Clostridium difficile*, and Extended-Spectrum-Beta-Lactamase- (ESBL-) producing Enterobacteriaceae [1–4]. The plasmid encoded cefotaximase enzymes CTX-M and the New Delhi metallo-beta-lactamase-1 (NDM-1) represent two excellent examples of the ESBL and carbapenemase that have been rapidly and globally disseminated. In this special issue, M. Elouennass et al. report on high rates of ESBL-producing Enterobacteriaceae and the emergence of carbapenemase-producing isolates in Morocco. The boom in medical tourism has seen patients from developed nations taking up low-cost private medical care in developing countries. This inevitably provides ample opportunity for clinically important AMR pathogens to be acquired and disseminated across geographical borders. A recent example is NDM-1 which was first described in an isolate from a Swedish patient who had previously been hospitalized in New Delhi, India [5]. NDM-1-producing isolates have subsequently been reported across several continents often detected in patients with history of recent medical care in the Indian subcontinent [6]. Preventive measures against nosocomial transmission of AMR pathogens include hand hygiene, environmental decontamination as well as screening and cohorting of patients. Data from the study by J. C. Catano et al., presented in this issue, identifies the diversity of areas of bacterial contamination in a tertiary healthcare setting in a developing country. As stated by the authors “these bacterial reservoirs are a plausible source of infections for patients” and it indicates the need for further research to evaluate strategies for minimizing risk of transmission to patients. 

In recognition of the global threat of the emergence and spread of multidrug-resistant bacteria worldwide, the World Health Organization (WHO) selected combating antimicrobial resistance as the theme for World Health Day 2011, issuing an international call for concerted effort geared towards halting the spread of antimicrobial resistance and recommending a six-point policy package for governments [7]. The fourth policy statement is to “regulate and promote rational use of medicines, including in animal husbandry, and ensure proper patient care.” Antimicrobial stewardship interventions promote judicious use of antibiotics and are critical in reducing emergence of resistance. In this issue, S. J. Patel et al. describe the development of an audit and feedback intervention based on the principles of the “model of actionable feedback” as well as the challenges to its implementation in a Neonatal Intensive Care Unit. 

In a seminal review in science in January 2000, Daszak, Cunninghamm and Hyatt underscored the “interaction with zoonotic pathogens within a host-parasite continuum between wildlife, domestic animals and human population” [8]. What we contend here is that as microorganisms can move freely in the diverse environments, so therefore can antibiotic resistance move without borders among humans, animals, and plants. The challenge posed by the emergence/reemergence of pathogenic microorganism from such interplay between humans, animals, and the environment is so significant that in 2009 the Royal Society issued a statement (RS Policy Document 2/09) pleading a “holistic approach” to infectious diseases in order to reach a “greater synergy between human and animal health sectors” [9]. There is widespread use of antibiotics such as glycopeptides and fluoroquinolones in animal husbandry with majority of these (estimates up to 90%) being administered not to treat infections but more controversially as growth enhancers. Section  4d of the aforementioned WHO policy statement specifically calls for reduction of use of antimicrobials in food-producing animals [7]. In 2003 The Lancet hosted an exceptionally appealing forum on “People, animals and antibiotic resistance” in which Dr. Wegener of the Danish veterinary institute reported on the ban on avoparcin (a glycopeptide), virginiamycin (streptogramin) tylosin, and spiramycin (macrolides) as growth promoters in the European Union [10]. However, as the use of antibiotics in food animals and in agriculture continues in other jurisdictions, the potential for clinically relevant pathogens gaining resistance markers from resistant strains selected in the environment remains a real threat. Indeed last year, an international research team reported that strains of *Enterococcus faecalis* isolated from pigs corresponded to a clone disseminated in several hospitals in Italy, Spain, and Portugal, harboring the same indistinguishable 100 kb mosaic plasmid—perhaps unequivocal evidence of trafficking of resistance genes in the environment between food animals and humans [11]. Of greater concern is that these genes (whether they originated before or after the EU ban) are now firmly established in the healthcare setting. The mechanisms, patterns, and clinical implications of emergence of resistance to glycopeptides and fluoroquinolones are presented in two in-depth reviews in this issue. Antibiotics from these two classes have been used extensively in animal husbandry, and findings from these reviews show that the WHO call is timely. 

The WHO recommends strengthening surveillance and laboratory capacity as the second policy statement for combating antimicrobial resistance [7]. The significant role played by international travel in the spread of multidrug-resistant bacteria urgently calls for a rapid and robust detection of these clinically important bacteria through functional surveillance systems. Most reports of importation of multidrug-resistant clinically important bacteria by returning travelers are from the industrialized countries with well-equipped clinical microbiology laboratory for rapid detection of new resistance mechanisms. Sadly, however, most developing countries where the multidrug-resistant bacteria successfully emerge and spread do not have functional clinical microbiology laboratories and the capacity for detecting bacteria with the novel resistance mechanisms. Disturbingly, developing countries are still plagued by inappropriate sewage systems, poor healthcare services, and severe overcrowding which together with unregulated use of antimicrobial agents favor the emergence and spread of multidrug-resistant bacteria. In this issue, P. Bhatter et al., provide us with a snapshot of the challenges to the public health system of two emerging economies (China and India) as they face the realities of tuberculosis control. The lessons learnt may serve as models for other developing countries. As recommended by the WHO, there is an urgent need for investment in global antimicrobial resistance surveillance systems, especially in developing countries to ensure prompt detection of emerging multidrug-resistant bacteria with novel clinically important resistance before their worldwide dissemination. 

Another important concern that should deserve more attention is the problem of global spread of antifungal resistance. Indeed, no paper related to antifungal resistance was received for peer review for this issue. As noticeably stated in a recent editorial in Science, billions of patients are suffering from fungal infections, caused by over 600 different fungal species [12]. Those previously considered not clinically relevant like *Fusarium, Malassezia, Paecilomyces, and Penicillium marneffei* are emerging as important pathogens associated with significant morbidity and mortality. No vaccine is available to fight these infections, diagnostic tests are cumbersome, and rapid tests, such as the recent *β*-1-3D glucan assay or galactomannan, are lacking specificity and are not affordable in many laboratories. Delay in the diagnosis and initiation of treatment of fungal infection can have devastating consequences. The available drugs, such as Amphotericin B, are extremely toxic (or the less toxic formulations are exceedingly expensive); the azoles show pharmacokinetic, pharmacodynamics, and resistance problems, thus resulting in increasing misuse of the echinocandins in empirical therapy. There is some evidence that at least in *Aspergillus fumigatus* the observed azole resistance might be related to the widespread use of fungicide in the environment especially in agriculture where azoles (such as fenbuconazole and propiconazole) are commonly used for plant protection. It is evident that the fact that molecules used in agriculture are different from those used in humans is in respect to the problem of emergence of resistance totally irrelevant. Extremely well-documented molecular investigations were presented by Verweij et al. [13]. The authors found that the *Aspergillus fumigatus* multi azole resistance which emerged in the Netherlands since 1999, with 6–13% of patients harboring the isolate, became resistant through a single resistance molecular mechanism represented by a substitution at codon 98 of cyp31A and a 34 bp tandem repeat in the gene promoter region. This mutation was detected in 99% of isolates and strains found in the soil and compost were genetically related. The bad news is that reports of this mutation TR/L98H have emerged from other European Countries (Belgium, France, Norway, Spain, and the UK) indicating spread of the resistance trait. Future implications with tremendous economic impact of this discovery are extremely important for the public health. Most likely, as done for antibiotics in food animals, regulations will be needed to restrict the use of antifungal agents of selected classes in agriculture, although preventing the spread of the current resistance might well prove to be a mission impossible.
